# Ethical trade-offs in AI for mental health

**DOI:** 10.3389/fpsyt.2024.1407562

**Published:** 2024-08-29

**Authors:** Sune Holm

**Affiliations:** Department of Food and Resource Economics, University of Copenhagen, Frederiksberg, Denmark

**Keywords:** AI, psychiatry, diagnosis, mental health, decision-making, fairness, explainability

## Abstract

It is expected that machine learning algorithms will enable better diagnosis, prognosis, and treatment in psychiatry. A central argument for deploying algorithmic methods in clinical decision-making in psychiatry is that they may enable not only faster and more accurate clinical judgments but also that they may provide a more objective foundation for clinical decisions. This article argues that the outputs of algorithms are never objective in the sense of being unaffected by human values and possibly biased choices. And it suggests that the best way to approach this is to ensure awareness of and transparency about the ethical trade-offs that must be made when developing an algorithm for mental health.

## 1 Introduction

AI is coming to psychiatry. The expectation is that machine learning (ML) algorithms will help improve diagnosis, prognosis, and treatment (1). Such algorithms have already shown expert-level accuracy in detecting medical conditions such as eye diseases (2), kinds of cancer (3), and pulmonary conditions (4) to mention a few. ML algorithms can also provide accurate estimates of the probability that a patient has an outcome of interest for mental health, e.g. of whether the patient will transition to psychosis (5). The average accuracy of ML algorithms in psychiatry has been estimated to be around 80 percent and improving (6). This level of accuracy would seem to be a boon to psychiatry which struggles with low predictive accuracy (7). Moreover, the development of algorithms focusing on mental health and psychiatric disorders is accelerating. For example, the yearly number of publications of algorithms for predicting depression more than doubled from 2013–2017 (6).

The comparatively high predictive accuracy of algorithms has been recognized for more than half a century. In 1954 Meehl (8) argued that statistical reasoning should play a more dominant role in clinical decision-making. In 1970 Sines (9) reviewed studies comparing statistical/actuarial and clinical methods for making predictions in psychopathology and concluded that “the actuarial predictions were found to exceed or at least equal the accuracy of clinical predictions” (9, 142). Dawes et al. (10) reconfirms this conclusion. Current studies of ML algorithms in medicine and psychiatry yet again shows that statistics-based methods can achieve expert-level accuracy.

A central argument for deploying algorithmic methods in clinical decision-making in psychiatry is that they may “enhance the speed, accuracy and objectivity” of clinical decisions (7, 172). Thus, using “objective and automated methods” for clinical decisions in psychiatry (11, 2) is expected to improve the highly subjective nature of decision-making in psychiatry, which in large part relies on the clinician-patient communication.

However, one must be careful when describing ML algorithms as objective. An algorithm may be said to be more objective than a clinician in the sense that training and test datasets, algorithm type, performance, and other factors can be made public for all to see. Still, characterizing an algorithm as objective signals that the algorithm’s output is not influenced by human values and biases. Being data-driven, the algorithm may be thought to simply look at the facts, derive a predictive pattern, and produce a prediction with no room for human bias to creep into the process. In fact, studies show that 40 percent of Americans consider it possible to produce algorithms which are objective in the sense of being free from human biases (12). In other words, there is a strong association between algorithmic predictions and value-neutrality. Algorithmic outputs are not influenced by human values. They just consider the facts and produce their predictions.

This article aims to highlight several ways in which algorithms incorporate choices and assumptions that reflect ethical trade-offs - notions of what is good and bad, right and wrong, fair and unfair. That is not to say that using algorithms might not improve on current methods of clinical psychiatry. There are aspects of algorithmic predictions which may indeed be thought to make predictions more accurate, uniform, reliable, and less prone to human biases. However, it is important also to explicate how predictive algorithms for psychiatry will be shaped by judgments that, perhaps unreflectively, invoke ethical values and trade-offs.

In this article I characterize these tradeoffs so that patients and psychiatrists are not lured into thinking that ML algorithms simply reflect the facts independently of any value-laden decisions. Hopefully the framework outlined may facilitate clarification and discussion of the value assumptions informing predictive algorithms which are candidates for being used in psychiatry.

There is a plethora of AI tools which may enter clinical practice. However, for the purpose of this analysis, I will focus on the prediction and diagnosis of mental disorders. I illustrate the considerations in relation to a hypothetical case of the development and use of an algorithm for predicting major depressive disorder (MDD) in primary care. Such use does not seem wholly unrealistic. Depression is a frequent and costly condition, and in most cases the initial diagnosis is made by general practitioners. However, as with other psychiatric disorders, you cannot just take a blood test and get a reliable determination of whether the condition is present. MDD comes in many guises and degrees making its diagnosis a complex and challenging affair, in particular in primary care, where physicians’ diagnostic accuracy tends to be low with general practitioners failing to make correct diagnosis in about 50 percent of cases (13).

## 2 Five ethical trade-offs

In this section I present and discuss five decision points in the development of a ML algorithm for MDD prediction which reflect ethical values.

### 2.1 What is the problem? Why use an algorithm?

Generally, the traditional methods of diagnosis rely heavily on interviews and questionnaires. There are several reasons why it might be attractive for patients and practitioners alike to welcome algorithmic diagnostic support. First, the traditional methods are time-consuming and labor-intensive. Moreover, their accuracy is highly dependent on the practitioner’s personal experience including not only years of experience but also the variety of patients to which the practitioner has been exposed in the past. Obviously younger practitioners must rely on less experience than more seasoned ones. In addition, practitioners will likely be influenced by some form of bias regardless of their experience and genuine attempt to rely only on relevant information. Finally, patients will also display personal differences when it comes to their ability and willingness to reveal information, e.g., due to fears of stigmatization. An algorithm applied, e.g., across the country may improve on this situation by providing more uniform assessments of patients based on empirically established patterns in much larger and more varied dataset than any individual practitioner can acquire and analyze on their own. In turn this may not only improve accuracy but also prevent the practitioner from missing out on relevant symptoms.

While these are some of the ways in which algorithmic support may improve on current methods, the choice to deploy an algorithm to classify patients with respect to some outcome does not take place in a vacuum. Opting for deploying a predictive algorithm will be guided by some goal that is assumed to be best achieved by better prediction. Thus, the first decision point concerns the identification and characterization of the goal of using the algorithm. What sort of problem is the algorithm supposed to help solve?

In our case of MDD prediction, there are many different goals that one might seek to achieve by introducing algorithmic MDD prediction in primary care. One might have as a goal to improve diagnostic accuracy. Or to achieve the same level of accuracy for less money. Other aims could be to address problems of over- and underdiagnosis or to address biases against certain patient groups among diagnosticians.

A decision to look to a predictive algorithm will rely on some goal or other, which provides the initial justification for investing in algorithmic prediction. The way the goal is stated determines the alternative courses of action and investment that will be competing with the algorithmic solution. Goal setting is clearly a value-laden activity. The goal one chooses to pursue expresses a notion about what one takes to be valuable states of affairs. Moreover, there might well be disagreement about whether a goal is indeed worth pursuing, and even if there is agreement about the goal, there might be disagreement about whether investing in an algorithmic approach is the best way to achieve the goal.

To illustrate, there might be agreement that it is important to improve diagnostic accuracy of MDD in primary care. One way to do so is to develop and implement algorithmic prediction tools. However, there are other ways in which this goal could be achieved. One alternative could be to invest in better education and training of general practitioners, or in providing them with better feedback on the diagnoses they make enabling them to learn from their past diagnoses. And if the problem to be solved is that men are underdiagnosed with MDD, an alternative way of handling the problem could be to improve physicians’ awareness of possible biases in diagnostic reasoning about male patients. In either case, the decision to invest in development and implementation of algorithmic MDD prediction is in effect to suggest that it is a better solution to a problem than alternatives. And as such the choice can be described as reflecting an ethical tradeoff: Assuming one cannot do both, it is better to invest in developing and implementing an algorithm than in better and targeted education of general practitioners.

### 2.2 Which data?

The quality of an algorithm for predicting MDD is dependent on the data available for training. This is because the training dataset provides the algorithm with a representation of the world in which it is going to be used – the world according to the data (14). Thus, the choice of training data is a choice about what representation of reality the algorithm is going to learn from. Notably, the way a training dataset is compiled will reflect value judgments, judgments about what makes the dataset good (enough) for the purpose of training the algorithm to predict the outcome of interest. Thus, “the data,” do not provide a representation of reality which is independent of human values and interests. When it comes to determining what to include in a training dataset several value-laden considerations will come into play.

To illustrate this, consider a team of algorithm developers with a fixed budget who must decide on how to construct their training dataset (15). How should they spend the data budget? Assuming that the real-world population consists of 50 percent men and 50 percent women, should they aim for a dataset that has equal representation of men and women? Perhaps it is more expensive for them to acquire depression diagnosis data about men. Thus, ensuring equal representation will result in a smaller dataset than allowing for a larger proportion of women.

And what sort of considerations would support one or the other decision? Perhaps the overall accuracy of the algorithm will be higher by allowing for an imbalance with respect to men and women. However, the comparative improvement of accuracy would be due to improvement for women. Prioritizing acquiring more data about men would on the other hand not achieve as high overall accuracy. However, it would ensure that the algorithm achieved almost equal accuracy rates for men and women.

In addition to considerations about representativeness such as those just described, another important decision concerns whether the data to be used are structured. In case they are, there is a question about which categories should be included. For example, there has been some debate about whether protected categories such as race and gender should be made available for the algorithm during training, as well as whether gender should be given a binary categorization. Again, I do not claim that there is a single correct way to construct a dataset. My point is that a dataset, the way the world is represented to the algorithm, is a construction based on several value-laden decisions concerning what in the present context makes for a good dataset.

### 2.3 Fairness

Problems of bias and fairness may arise for several reasons. Some important ones include skewed datasets used for training and testing (16), choice of proxy variable (17), and the use context of the algorithm, e.g., on a population that differs from the population represented in the training and testing data.

In relation to our focus on the prediction and diagnosis of mental disorders a key issue concerns how to fairly distribute prediction errors across salient groups such as protected groups referred to in relation to discrimination legislation. The issue became top of the agenda when an algorithm for predicting recidivism widely used in the criminal justice system in the United States was criticized for being biased against Black defendants (18). The problem identified by Angwin et al. (18) was that COMPAS, as the algorithm is called, seemed to have very different error rates for Black and White defendants. Thus, it was twice as likely to falsely flag a Black defendant as high risk of re-offending if released before trial as compared to a white defendant. And it was twice as likely to falsely flag a White defendant as low risk of re-offending if released before trial as compared to a Black defendant.

A wide range of algorithmic fairness definitions has been explored in detail since Angwin et al. (18). A key upshot of this literature is that there are several plausible candidates for algorithmic fairness and that, as a matter of mathematics, not all plausible candidate definitions of algorithmic fairness can be met simultaneously in ordinary circumstances (e.g., 19, 20). Tradeoffs must be made. To see this consider a situation in which one wants the MDD algorithm to have the same sensitivity and specificity (or true positive and true negative rate) for men and women. The ratio of true positive predictions to the total number of predictions about patients who in fact have MDD should be the same for male and female patients. And the ratio of true negative predictions about patients who are in fact negative for MDD should be the same for men and women. In ordinary circumstances, the frequency of MDD will differ for the male and female population. However, if this is the case, then achieving equal sensitivity and specificity can only be achieved if some men and women, who are estimated to have the same probability of suffering MDD, do not receive the same prediction. This is because to achieve equal sensitivity and specificity, the algorithm will have to apply different classification thresholds to men and women.

What transpires is that when designing an algorithm for MDD prediction, its performance will tend to differ with respect to protected groups such as groups defined by gender or race. However, how it differs is a design choice. If one decides to set up the algorithm to produce equal error rates for men and women one also accepts that there will be men and women who will not receive the same prediction despite the fact that they are estimated to be equally likely to suffer MDD. Alternatively, one can decide to apply the same threshold for when the algorithm should make a positive prediction of MDD for all individuals. However, in that case one also accepts that error rates might be very different for protected groups. The disparity in error rates will in turn affect the burdens imposed on these groups from erroneous predictions. Thus, the design of an algorithm will reflect a decision about the appropriate distribution of prediction errors across groups.^1^

### 2.4 Explainability

The main selling point of predictive ML algorithms is their comparatively high accuracy. The most accurate types of algorithms are often described as “black boxes.” This characterization aims to convey the fact that deep neural networks and other types of highly accurate ML algorithms are too complex for humans to be able to explain how they arrive at an output from an input. In response to the black box nature of algorithms, research on “Explainable AI” or XAI has surfaced with researchers trying to develop tools that may help users understand why an individual prediction was produced based on an input from a patient.

The black box nature of ML algorithms reflects their central strength. The complexity of the algorithm is not limited by human capacities for finding predictive patterns in a dataset and manually transforming the pattern into a mathematical model. The flipside is that ML models go beyond what humans can comprehend and explain. An alternative to ML algorithms is algorithms which are interpretable. Such models are simpler and perhaps less accurate. However, they have the advantage of being such that humans can understand and explain how they arrive at a prediction based on an input. A linear function with one or two predictive variables may not be as accurate as a complex deep neural network but it is easy for humans to grasp how the values of the input variables produce the prediction.

The development of an algorithm for predicting MDD thus involves an important tradeoff between the improvement of accuracy that may be achieved by a highly complex algorithm versus the possibility that humans can understand why the algorithm generated a particular output from an input. In the context of decision-making in psychiatry explainability would seem to be of great importance to ensure that algorithmic outputs can be trusted and acted upon.^2^

### 2.5 Information presentation

An algorithm to be used in clinical practice must convey information to its user. Focusing on cases of prediction and diagnosis, the assumption has been that an MDD prediction algorithm would provide the user with a binary output: positive or negative for MDD. However, it is by no means obvious that this is the proper way to present the output of the algorithm to the user. To begin with, a binary classification of patients will be based on a predetermined threshold such that patients whose risk score is at or above the threshold will be classified as positive for MDD and those scoring below the threshold are classified as negative. However, the information provided by the algorithm could also be the risk score of the patient. This would leave more room for the user to decide whether to classify the patient as MDD.

At this point it also becomes important to keep in mind that algorithms rely on statistical reasoning when they assign a risk score to an individual patient. The sort of inference that the algorithm in effect makes when it assigns a risk score to a patient is the following:

1. The proportion of patients with features X who suffer MDD = n.
2. This patient has features X.
3. Hence the probability that this patient suffers MDD = n.

There is a fundamental issue about the validity of this kind of inference from the frequency of, say, MDD in a reference group to the risk that an individual member of the reference group has MDD (24). There can easily be features of the individual member which make her much more or much less likely to have MDD than the probability that a randomly drawn member of the reference group suffers MDD. To bring this out, one could require that this was made explicit in the way the algorithm presented its finding: Patient A belongs to a group of patients of which n/100 are positive for MDD.

## 3 Concluding remarks

ML algorithms are showing promising results for prediction of psychiatric conditions. In this article I have argued that psychiatrists should be wary of claims that ML algorithms are objective in the sense of not being influenced by human values and biases. At several stages in the development of a predictive algorithm decisions must be made which will reflect value judgments.

## Data availability statement

The original contributions presented in the study are included in the article/supplementary material. Further inquiries can be directed to the corresponding author.

## Author contributions

SH: Conceptualization, Writing – original draft, Writing – review & editing.

## Footnotes

1 There is a large and growing literature on ways to measure and detect fair use of AI, such as tests that assess whether the results are equivalent for different groups of people. For a recent overview considering “how to measure and assess fairness and how to mitigate bias in models, when necessary,” see Castelnovo et al. (21, 1). Holm (22) may provide a way into the philosophical discussion of algorithmic fairness.

2 While I have outlined how explainability may have to be traded off against accuracy, it should be noted that this does not exclude that improvements in explainability can benefit the clinical process. One study indicating this is Tonekaboni et al. (23), which provides insights into which classes of explanations clinicians find to be “most relevant and crucial for effective translation to clinical practice” (23, 1).
